# Lymph Node Dissection in Upper Tract Urothelial Carcinoma: Current Status and Future Perspectives

**DOI:** 10.1007/s11912-023-01460-y

**Published:** 2023-10-06

**Authors:** Andrzej Dłubak, Jakub Karwacki, Katarzyna Logoń, Paulina Tomecka, Kinga Brawańska, Wojciech Krajewski, Tomasz Szydełko, Bartosz Małkiewicz

**Affiliations:** Department of Minimally Invasive and Robotic Urology, University Center of Excellence in Urology, Wroclaw Medical University, 50-556, Wroclaw, Poland

**Keywords:** Upper tract urothelial carcinoma, Lymphadenectomy, Lymph nodes, Oncologic staging, Urologic malignancies

## Abstract

**Purpose of Review:**

This narrative review aims to evaluate the role of lymph node dissection (LND) in upper tract urothelial carcinoma (UTUC) and its implications for staging and management outcomes, as well as future perspectives.

**Recent Findings:**

Multiple studies have demonstrated the limitations of conventional imaging techniques in accurately localizing lymph node metastasis (LNM) in UTUC. While 18F-fluorodeoxyglucose positron emission tomography with computed tomography (18FDG-PET/CT) shows promise for preoperative LNM detection, its specificity is low. Alternative methods such as choline PET/CT and sentinel lymph node detection are under consideration but require further investigation. Additionally, various preoperative factors associated with LNM hold potential for predicting nodal involvement, thereby improving nodal staging and oncologic outcomes of LND. Several surgical approaches, including segmental ureterectomy and robot-assisted nephroureterectomy, provide a possibility for LND, while minimizing morbidity.

**Summary:**

LND remains the primary nodal staging tool for UTUC, but its therapeutic benefit is still uncertain. Advances in imaging techniques and preoperative risk assessment show promise in improving LNM detection. Further research and multi-center studies are needed to comprehensively assess the advantages and limitations of LND in UTUC, as well as the long-term outcomes of alternative staging and treatment strategies.

## Introduction

Upper tract urothelial carcinoma (UTUC) is a rare malignancy originating from the urothelial lining of the urinary tract [1]. It accounts for 5–10% of all urothelial cancers, with an estimated annual incidence in Western countries of 1–2 cases per 100,000 people [2•, 3••]. Despite its low incidence, UTUC often presents with lymph node (LN) metastasis, making diagnosis and management challenging. Approximately 60% of UTUC cases are invasive at the time of diagnosis, highlighting the importance of accurate staging [4].

Radical nephroureterectomy (RNU) is the gold standard treatment in non-metastatic UTUC [3••, 4, 5]. It provides durable local control and high cancer-specific survival (CSS) rates [4, 6•]. However, in low-risk UTUC, kidney-sparing surgery is considered a viable alternative, as it demonstrates comparable survival outcomes to RNU in these patients [7]. For patients with nodal involvement (pN +), current guidelines advocate RNU followed by adjuvant platinum-based chemotherapy (ChT) [3••]. The role of lymph node dissection (LND) in UTUC is still under investigation, given the lack of cohesive guidelines and limited therapeutic value [3••, 8–10]. LN involvement is a predictor of lower CSS, and overall survival (OS), making LND crucial for accurate nodal staging and identification of individuals who may benefit from adjuvant therapy [11, 12••, 13–17]. While there is ongoing debate regarding the efficacy of LND in UTUC, with studies showing conflicting results, its potential benefits remain a subject of interest [4, 11, 18–20, 21•, 22].

This study aims to review the status and future perspectives of LND in UTUC, focusing on the implications of LN invasion, the challenges in establishing an optimal LND template, and the potential survival benefits associated with the number of LNs removed. We aim to provide valuable insights for improving diagnostic accuracy and refining therapeutic approaches in the management of UTUC.

## Methods

Databases, including PubMed/Medline and Embase, were searched using various combinations of keywords such as “UTUC,” “upper tract urothelial carcinoma,” “lymphadenectomy,” and “lymph node dissection.” Only English-language articles published between January 1980 and April 2023 were included. A total of 178 papers were found, of which 364 were selected as sources for the subsequent review. Original articles, systematic and narrative reviews, meta-analyses, and editorials were selected based on their clinical relevance. Additionally, the references cited in the selected studies were reviewed to identify and include significant papers that were initially excluded from our primary search.

## Prognostic Factors for Nodal Involvement

Several prognostic factors for nodal involvement in UTUC have been described. Postoperative pathological parameters include tumor size (> 4 cm), stage, grade, and multifocality, extensive tumor necrosis, location in the renal pelvis (RP) and on the left side, lymphovascular invasion (LVI), and perineural invasion (PNI) [23–27, 28•]. Operative factors involve positive surgical margins and the number of LNs removed during LND, with a probability of 24% for patients with one LN removed and peaking at 31% when around 15 LNs were removed [17]. Interestingly, a study by Deuker et al. found that increasing age was correlated with lower rates of lymph node metastasis (LNM) in women but not in men, while a study by Inokuchi et al. identified older age as a predictive factor for LN involvement in both sexes [29, 30]. Elevated preoperative levels of fibrinogen, cystatin-C, C-reactive protein (CRP), and neutrophil-to-lymphocyte ratio (NLR) (> 2.7) are associated with LN involvement, as well as low albumin-globulin ratio (AGR) (< 1.45), and preoperative anemia [31–36]. The summary of the prognostic factors for LNM is provided in Table 1.

**Table 1:** Overview of prognostic factors for nodal involvement in UTUC

Type	Prognostic factor	
Operative and pathological	Extensive tumor necrosis, LVI, location in RP and on the left side, number of LNs removed during LND, PNI, positive surgical margins, presence of local LN infiltration, tumor grade, tumor multifocality, tumor size > 4 cm, tumor stage	
Clinical	Older age	
	Elevated preoperative serum levels	• CRP, cystatin-C, fibrinogen, NLR
	Lowered preoperative serum levels	AGR, hemoglobin

*LVI* lymphovascular invasion, *RP* renal pelvis, *LNs* lymph nodes, *LND* lymph node dissection, *PNI* perineural invasion, *CRP* C-reactive protein, *NLR* neutrophil-to-lymphocyte ratio, *AGR* albumin-globulin ratio

## LND as A Diagnostic Tool

Computed tomography (CT) imaging following RNU has low sensitivity in identifying LNM, and therefore, it should not be relied upon to determine the need for LND [37]. Thus, LND is recommended for all patients undergoing RNU [38]. A recent systematic review confirms the significant staging benefits of lymphadenectomy [39]. If suspicious LNs are detected in CT, extended LND, encompassing the identified regions, should be performed [37].

The most commonly used staging system for UTUC is the American Joint Committee on Cancer (AJCC) stage grouping system [40]. It includes tumor stage, nodal stage, and the presence of metastases to predict patient prognosis and guide management decisions [41]. UTUC has four stages, and LNM classifies it as stage IV, which includes metastatic (M1) and locally advanced (T4) disease as well [40]. The AJCC stage grouping system for UTUC remains almost the same in the 6th, 7th, and 8th edition, while the stage grouping system for urothelial carcinoma of the bladder (UCB) has changed noticeably. In the 8th edition for UCB, T4 and N + stages were separated from M1 stage, while in the 8th edition for UTUC, this aspect remained unchanged [42, 43]. Several studies recommended that stage IV UTUC also should be modified [44•, 45]. A recent study by Abdel-Rahman suggests dividing it into nonmetastatic (T4 and N +) and metastatic disease (M1) subcategories. The results imply that it would improve the prognostic utility compared to the current system [45]. The same modification was suggested in a study by Li et al. as a one proposition. Second proposed modification was based on tumor grade and included dividing IV stage into low grade (T4, N + , M1, G1–2) and high grade (T4, N + , M1, G3–4) [44•]. The current staging system and the suggested changes are shown in Table 2.

**Table 2:** Current AJCC staging system and proposed modifications

Stage	AJCC 6th, 7th, and 8th editions [10, 40, 46, 47]	Modification proposed in studies by Abdel-Rahman and Li et al. [44•, 45]	Modification proposed in the study by Li et al. [44•]
I	T1N0M0	T1N0M0	T1N0M0
II	T2N0M0	T2N0M0	T2N0M0
III	T3N0M0	T3N0M0	T3N0M0
IV	T4 or N + or M1	IVA: T4 or N + M0 IVB: any T any N M1	Low grade: T4 or N + or M1; G1–2 High grade: T4 or N + or M1; G3–4

*AJCC* American Joint Committee on Cancer

## Oncological Outcomes

The therapeutic role of LND in RNU in UTUC patients remains questionable [10, 18, 48, 49•]. Several systematic reviews and meta-analyses investigated oncological outcomes of lymphadenectomy in UTUC [20, 50–52]. However, a challenge in achieving unbiased comparison arises because patients undergoing LND typically exhibit more advanced tumor stages and grades [12••]. A meta-analysis by Yang et al. was one of the first to assess survival rates in LND and non-LND groups. There was no significant difference in survival rates between LND and non-LND. Nevertheless, in the muscle-invasive patients, the LND group showed higher CSS (HR: 2.19; 95% CI: 1.26–3.80; *p* = 0.005) [50]. The systematic review be Dominguez-Escrig et al. pointed that LND could be most beneficial to patients with ≥ pT2 [52]. Guo et al. found no difference in CSS and recurrence-free survival (RFS) between pN0 and pNx groups, both in overall populations and in patients with muscle-invasive tumor [51]. The meta-analysis by Chan et al. showed no significant improvement in RFS (HR: 0.89; 95% CI: 0.41–1.92), CSS (HR: 0.89; 95% CI: 0.54–1.46), and OS (HR: 1.10; 95% CI: 0.93–1.30), but once again revealed that patients with advanced UTUC (pT2 and pT3) could benefit from LND. Omitting lymphadenectomy in these patients significantly worsened RFS (HR: 2.83; 95% CI: 1.72–4.66) [20].

Similar conclusions can be drawn from more recent publications as well [21•, 53•, 54•]. However, there are studies that point out therapeutic benefits of LND in UTUC. In the propensity score matching study by Ishiyama et al., researchers divided patients into two groups, one of which received complete LND, while the other had no or incomplete LND. CSS, OS, and metastasis-free survival (MFS) were significantly higher in the complete LND group (*p* < 0.05) [55••]. Another study by Kanno et al. revealed the estimated 5-year RFS was significantly higher in the LND group compared with that in the non-LND group (64.2% vs. 86.8%; *p* = 0.014) [56]. A recent study by Cui et al. showed that LND could be most beneficial in patients with tumors localized in the distal ureter (DU). Furthermore, the LND group was associated with higher RFS (27.0% vs. 18.3%;* p* = 0.044) and CSS (53.2 vs. 39.8%; *p* = 0.031) [57••].

In general, the advantage of LND may be more noteworthy for larger localized tumors [39]. The excision of a greater number of LNs was also associated with enhanced survival outcomes in patients with UTUC [58]. Extended LND involving the removal of four or more regional LNs may confer a benefit in terms of OS or CSS for patients in stages pT1–pT3 [20]. However, the LND template is likely to have a greater impact on patient survival than the number of LNs removed [10].

## Effect on Further Therapeutic Process

**Fig. 1:**
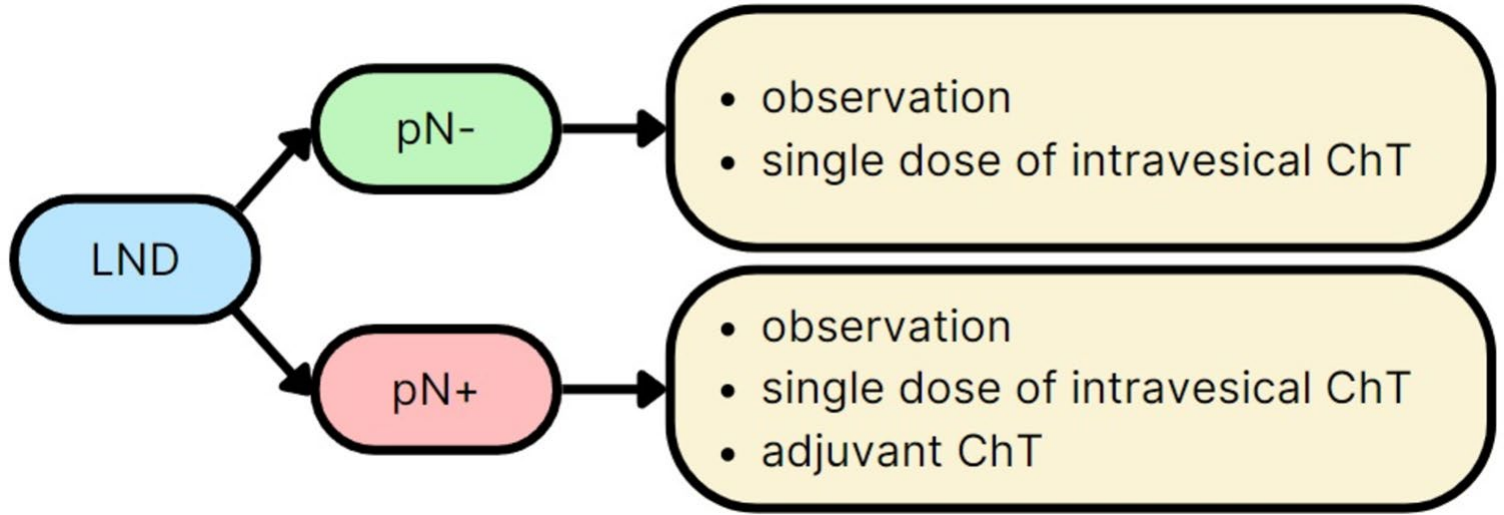
Influence of LND outcome on further therapeutic process

LND plays an important role in patient selection for adjuvant therapies after RNU [59]. Available studies indicate that gemcitabine-platinum combination ChT started within 90 days after RNU significantly improves DFS in patients with locally advanced UTUC [60•]. For metastatic urothelial carcinoma, a meta-analysis showed that cisplatin-based ChT, compared with carboplatin-based ChT, significantly increases the likelihood of both overall response and complete response [61]. Seisen et al. demonstrated an OS benefit from adjuvant platinum-based ChT in patients with pT3/T4 and/or pN + UTUC [62]. In case of non-progressive disease after platinum-based ChT, subsequent maintenance immunotherapy (avelumab) is recommended [64]. Patients positive for programmed death ligand 1 (PDL-1) and ineligible for cisplatin may receive immunotherapy (atezolizumab or pembrolizumab) [64]. Moreover, adjuvant radiotherapy has been suggested to control locoregional disease after surgical removal [65]. However, there is no clear benefit of such treatment after RNU [66]. In terms of the follow-up after RNU, the EAU guidelines indicate cystoscopy at 3 and 9 months, and then annually, for low-risk UTUC, as well as CT urography (CTU) once a year. For patients with high-risk UTUC, cystoscopy with cytology should be performed every 3 months for 2 years, then every 6 months for 2 years, and after this annually for 5 years, along with CTU [67]. However, a recent meta-analysis proposed a revision of the current guidelines regarding surveillance protocols. It suggests increase in frequency of imaging to semiannual until the 4th year after RNU [68••]. The management in line with current guidelines is outlined in Fig. 1.

## Lymphatic Drainage Patterns in UTUC

**Fig. 2:**
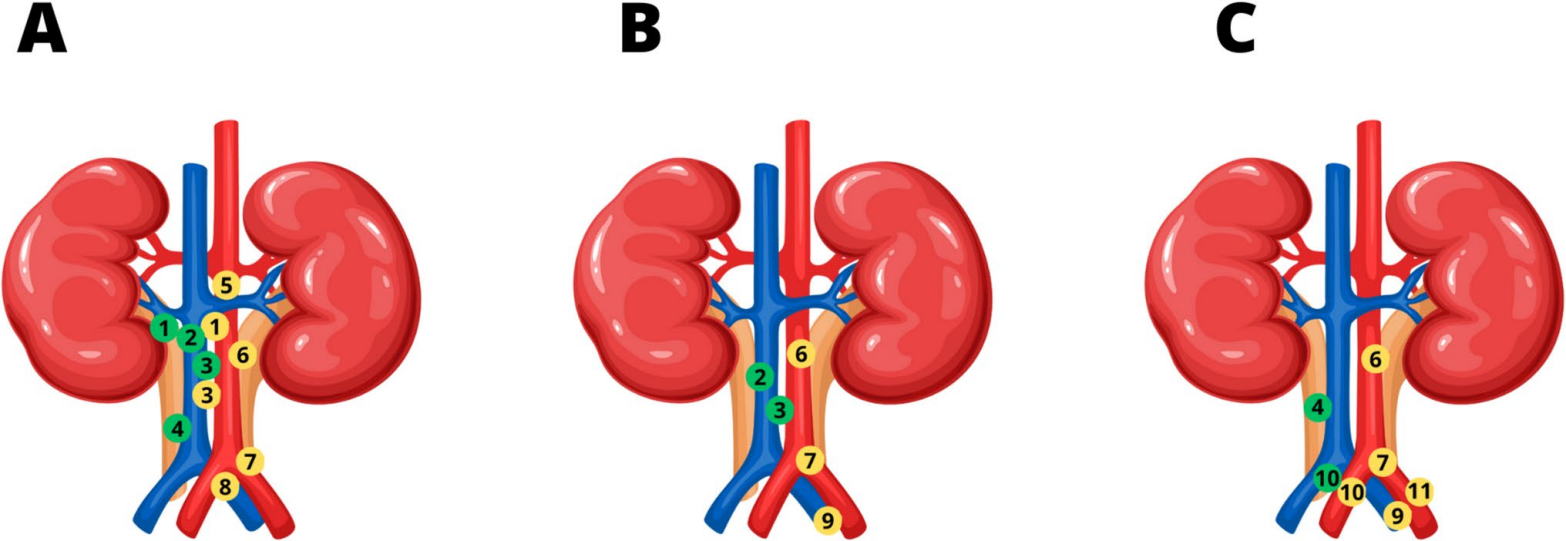
Regional nodal sites for the renal pelvis and the upper ureter (**A**), middle ureter (**B**), and distal ureter (**C**). *Yellow* affected nodal sites with primary tumor on the left side, *green* affected nodal sites with primary tumor on the right side. (1) Hilar, (2) retrocaval, (3) interaortocaval, (4) paracaval, (5) suprahilar, (6) paraaortic, (7) common iliac, (8) aortic bifurcation, (9) internal iliac, (10) presacral, (11) external iliac [73, 75]

LNs are the most common metastatic sites in UTUC; thus, understanding lymphatic drainage patterns is vital for establishing anatomical extent of LND [29, 30, 69–71]. The first mapping study on lymphatic drainage in UTUC was conducted by Akaza et al. in the 1980s [72]. They found that for tumors located in the upper half of the ureter (above the crossing with common iliac artery), regional LNs were the paraaortic (PA) and paracaval (PC) LNs. For tumors in the lower half of the ureter, intrapelvic LNs were considered regional. This study played a role in developing the TNM classification for UTUC. Kondo et al. expanded on this research and identified eight regions of LNM based on the primary tumor’s location [72, 73]. They included right/left RP, right/left upper ureter (UU), right/left middle ureter (MU), and right/left DU. The UU was the upper third of the ureter (superior to the inferior mesenteric artery), the MU was considered the middle third from the level of the inferior mesenteric artery to the crossing with the common iliac artery, and the DU below this crossing. For the right-sided RP, UU, and MU tumors, right renal hilar (RH), paracaval (PC), and retrocaval (RC) LNs were considered regional. Additionally, interaortocaval (IAC) LNs were included as regional LNs in the case of ureteral tumors. For cancers of the left RP, UU, and MU, the left RH and PA LNs were considered regional. For tumors of the DU, the ipsilateral common iliac (CI), external iliac (EI), obturator (Ob), and internal iliac (II) LNs were included as regional sites of LNM. It is worth noting that nodal involvement rate was lower in DU (8.3% for right and 13.3% for left) than in RP (30.6% and 24.2%), UU (33.3% and 0%), and MU (20.0% and 42.9%). In 2012, Kondo et al. updated their study and obtained the results similar to those from previous studies [74]. However, they suggested comprising IAC LNs as regional for tumors originating from the right RP, and the presacral nodes for DU tumors, despite the fact they accounted for only 14% LNM in right RP and 14% LNM in right DU (left DU was not mentioned). Hence, the authors advocated for including nodal sites at more than 10% risk of metastasis as regional LNs. Matin et al. conducted a similar study [75]. Right RP tumors had LNM to the RH (22.1%), PC (44.1%), RC (10.3%), and IAC (20.6%) regions. Right upper ureter tumors had LNM to RH (46.2%), PC (46.2%), and RC (7.7%) regions. There were no metastases to the right MU. Right DU tumors had LNM equally to PC and pelvic regions. Left RP tumors had LNM to RH (53.0%) and PA (31.0%) regions. There were also positive LNs in IAC (4%), suprahilar (1%), CI (1%), rectocrural (2%), and aortic bifurcation (1%) sites. The 7% of the landing sites were unspecified. Left UU tumors had LNM to RH (36.4%) and PA (63.6%) regions. Left MU tumors had LNM to PA (40%), CI (40%), and II (20%) regions. Left DU tumors had LNM to PA (33.3%), CI (33.3%), and EI and II (16.7% each) sites. IAC involvement from both sides as well as out-of-field LNM appeared to occur secondarily. Figure 2 depicts a visual representation of the regional lymphatic drainage based on the abovementioned studies.

## Anatomical Extent of Lymphadenectomy

LND extent can be either template-based LND or determined by the number of removed LNs [18]. Kondo et al. argued that the total number of removed LNs does not affect UTUC patients’ survival. They suggested that anatomical template-based dissection is more beneficial [76]. Subsequent studies supported these findings and advocated for either complete or incomplete LND, with complete LND involving the resection of all regional LNs [73, 76]. In a 2014 study, the authors once again supported template-based LND, although improved CSS and OS were observed only in patients with RP tumors [77]. However, there was a selection bias as the non-LND group primarily consisted of older patients with severe comorbidities.

The extent of LND has been described by the European Association of Urology (EAU), the National Comprehensive Cancer Network (NCCN), and the American Urological Association (AUA) and is shown in Table 3. The EAU template is based on three studies with only two providing specific templates [52, 75, 77]. Kondo et al. suggest resecting RH, PC, RC, and IAC for right-sided RP, UU, and MU tumors and RH and PA for left-sided RP, UU, and MU tumors. For DU tumors, the authors advocate for resection of ipsilateral CI, EI, II, and Ob LNs [77]. In the study by Matin et al., there is a separate template for MU tumors and the authors differentiate primarily excised LNs and additional dissection sites that could provide a greater rate of capturing possible LNMs [75]. For right-sided RP and UU tumors, primarily RH, PC, and RC and the addition of IAC LNs would increase the rate. For left-sided RP and UU tumors, primarily RH, PA, and adding IAC LNs would increase the rate. Right-sided MU tumors include IAC LNs, while adding PC and RC nodes would remove remaining LNMs. Left-sided MU tumors include PA LNs, while adding CI and II nodes would remove remaining LNMs. For right-sided DU tumors, primarily CI, EI, II, and Ob LNs were dissected, while adding PC nodes would remove remaining LNMs. For left-sided DU tumors, CI, EI, II, and Ob LNs were dissected, while adding PA nodes would remove remaining LNMs.

**Table 3:** Guidelines for indications and extent of LND

Guideline	Indications and the extent of LND		Reference
	Indications for LND	Extent of LND	
EAU	Template-based LND should be offered to patients who are scheduled for RNU for HR non-metastatic UTUC	In Kondo et al. study: • Right-sided/left-sided RP, UU, and MU tumors: RH, PC, RC, and IAC LNs/RH and PA LNs • Right-sided/left-sided DU tumors: ipsilateral CI, EI, Ob, and II LNs In Matin et al. study: • Right-sided/left-sided RP and UU tumors: primarily RP, PC, and RC LNs/RH and PA LNs, while adding IAC nodes on both sides would further increase the chance of capturing all LNMs • Right-sided/left-sided MU tumors: primarily IAC LNs/PA LNs, while adding PC and RC nodes/CI and II nodes would remove remaining LNMs • Right-sided/left-sided DU tumors: primarily ipsilateral CI, EI, II, Ob LNs, while adding PC nodes/PA nodes would remove remaining LNMs	[3••, 75, 77]
NCCN	LND is recommended for patients with high-grade upper genitourinary tract tumors	• Left-sided RP, UU, and MU tumors: regional LND should include the PA LNs from the RH to the aortic bifurcation. Most MU tumors will also include the CI, EI, Ob, and hypogastric LNs • Right-sided RP, UU, and MU tumors: regional LND should include the PC LNs from the RH to the inferior vena cava bifurcation. Most MU tumors will also include the CI, EI, Ob, and hypogastric LNs • DU tumors: regional LND should be performed and include the CI, EI, Ob, and hypogastric LNs	[89••]
AUA	LND may be performed in LR UTUC at the time RNU or ureterectomy. LND should be performed in HR UTUC at the time RNU or ureterectomy	• Tumors in the pyelocaliceal system: LNs of the ipsilateral great vessel extending from the RH to at least the inferior mesenteric artery • Tumors in the upper 2/3 of the ureter: LNs of the ipsilateral great vessel extending from the RH to the aortic bifurcation • Tumors in the distal 1/3 of the ureter: ipsilateral pelvic LND to include at minimum the Ob and EI nodal packets. II and CI nodal packets may be removed in the appropriate clinical setting. Limited data suggest cranial migration of LNM to the ipsilateral great vessels such that higher dissection may be considered in the appropriate clinical setting and per clinician judgment	[88••]

*EAU* The European Association of Urology, *NCCN* The National Comprehensive Cancer Network, *AUA* The American Urological Association, *LND* lymph node dissection, *RNU* radical nephroureterectomy, *NU* nephroureterectomy, *LNs* lymph nodes, *UTUC* upper tract urothelial carcinoma, *LR* low risk, *HR* high risk, *LNM* lymph node metastasis, *RP* renal pelvis, *UU* upper ureter, *MU* middle ureter, *DU* distal ureter, *RH* renal hilar, *PC* paracaval nodes, *RC* retrocaval nodes, *IAC* interaortocaval nodes, *PA* para-aortic nodes, *CI* common iliac nodes, *EI* external iliac nodes, *II* internal iliac nodes, *Ob* obturator nodes

The NCCN suggests resection of the PC LNs from the RH to the inferior vena cava bifurcation, and CI, EI, Ob, and hypogastric LNs in most MU tumors, and similarly for the left side PA LNs from the RH to the aortic bifurcation and CI, EI, Ob, and hypogastric LNs in most MU tumors. In DU tumors, ipsilateral CI, EI, Ob, and hypogastric LNs should be removed.

**Fig. 3:**
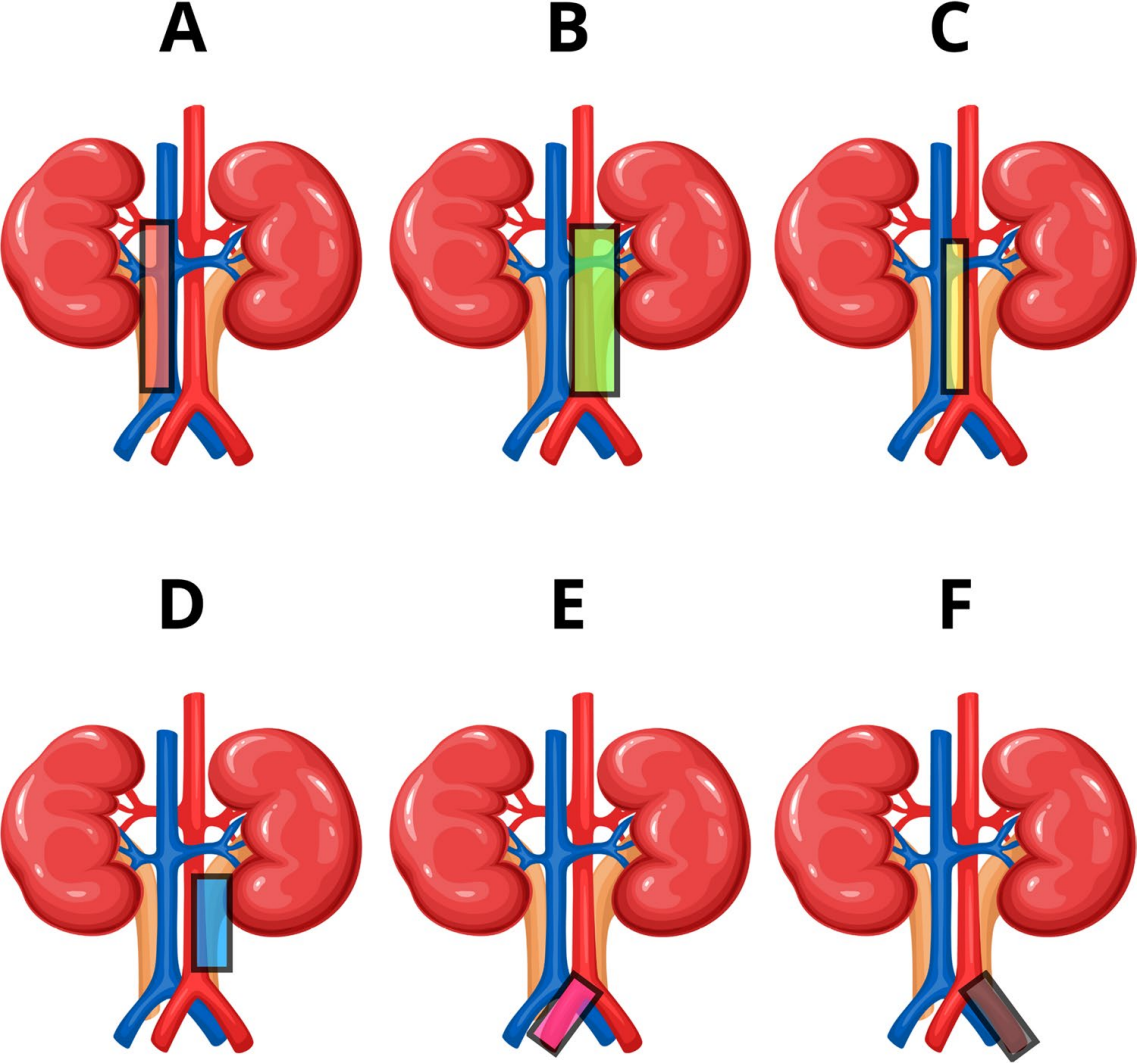
Anatomical extent of LND according to the Matin et al. study, and consistent with the Kondo et al. study [75, 77]. The two abovementioned studies were used by the EAU guidelines to demonstrate the extent of LND. **A**–**F** show the templates for LND based on primary tumor’s location. **A** Right renal pelvis and upper ureter (red: renal hilar, paracaval, and retrocaval LNs). **B** Left renal pelvis and upper ureter (green: renal hilar para-aortic LNs). **C** Right middle ureter (yellow: interaortocaval LNs). **D** Left middle ureter (blue: para-aortic LNs). **E** Right distal ureter (pink: common iliac, external iliac, internal iliac, obturator LNs). **F** Left distal ureter (gray: common iliac, external iliac, internal iliac, obturator LNs)

The AUA for tumors in the pyelocaliceal system suggests removing LNs of the ipsilateral great vessel extending from the RH to at least the inferior mesenteric artery, and for tumors in the upper 2/3 of the ureter LNs of the ipsilateral great vessel extending from the RH to the aortic bifurcation. For tumors in the distal 1/3 of the ureter ipsilateral pelvic, LND should include at minimum the Ob and EI LNs. II and CI may be removed in the appropriate clinical setting. The authors stated that limited data suggest cranial migration of LNM to the ipsilateral great vessels such that higher dissection may be considered in the appropriate clinical setting and per clinician judgment. Through the years, the studies have shown that in terms of the extent of LND the approach is incohesive and differs based on the institution and surgeon’s decision [15, 49•, 71, 73, 75, 78–83]. Figure 3 shows the anatomical extent of LND according to studies used in EAU guidelines and shows primarily excised regions.

## Complications of LND

Ishiyama compared complications using the Clavien-Dindo classification in the complete LND group and the no/incomplete LND group after matching (16.7% vs 20.0%;* p* = 0.7385) [55••]. Only lymphatic leakage complications differed significantly, with 4.76% in the complete LND group and 0% in the no/incomplete LND group (*p* = 0.0231). Other complications showed no significant differences. In the complete LND group, only one patient experienced high-grade (≥ 3) complication. Pearce et al. studied 16,619 patients undergoing RNU for urothelial carcinoma, including 2560 who received LND [84]. Intraoperative complications occurred in 4% of both groups. The LND group had a higher rate of postoperative complications (27 to 29%), but the difference was not significant (*p* = 0.4). However, multivariate analysis showed a 30% higher chance of postoperative complications in patients who underwent LND (OR: 1.3; 95% CI: 1.001–1.7;* p* = 0.049). Winer et al. found that increased LN yield was associated with increased odds of any complication within 30 days (OR: 1.18 [per 5 nodes]; 95% CI: 1.05–1.32;* p* = 0.004), but no increased risk of grade ≥ 3 complications [85]. Furthermore, extensive LND did not significantly increase operative time or estimated blood loss. The randomized study by Blom et al. showed no significant impact of LND extension on complication rate, morbidity, or mortality [86]. However, bleeding exceeding 1 L (9.4% and 6.5%), embolism (2.2% and 1.1%), and lymph fluid drainage (3.9% and 2.4%) occurred at a higher rate in the LND group. Kondo et al. conducted comparison of complications between the template-based LND group and the non-LND group [87]. Patients who underwent LND experienced more complications across all Clavien-Dindo grades, but the difference was not statistically significant. The LND group had a higher incidence of complications such as numbness in the thighs (2.6% and 0%) and lymphorrhea (5.2% and 1.1%). The numbness in the thighs could be associated with pelvic LND. The overall incidence of complications and grade ≥ 3 complications were 14.2% and 3.9% in the LND group, compared to 10.1% and 1.1% in the non-LND group. In their previous study, the authors demonstrated that operation time and intraoperative bleeding were greater in the LND groups (323 min and 288 min, 407 mL and 321 mL, respectively) [74]. The authors concluded that LND may slightly increase complications such as lymph fluid drainage and hemorrhage, but these complications do not significantly affect patients’ recovery after surgery.

## Current Guidelines

Table 3 provides an overview of the current guidelines. Presented recommendations are cohesive regarding the indications for LND in high-grade/high-risk UTUC. However, only the AUA guidelines mention LND for low-risk UTUC. The Memorial Sloan Kettering Cancer Center (MSKCC) and The National Institute for Health and Care Excellence (NICE) did not provide specific guidelines concerning LND in UTUC. In Table 3, low-risk UTUC was defined by low-grade biopsy and normal cytology and high-risk UTUC by high-grade biopsy or cytology with disease progression risk and pathologic stage T2 or greater [88••].

## Future Perspectives

Research suggests that certain laboratory tests and genetic markers can provide insights into the prognosis of UTUC and UBC [90, 91•, 92]. The non-invasive tests discussed in this section can be utilized to identify patients who are more likely to have cancer metastasis to the LNs. This, in turn, enables more accurate selection of patients who would benefit from LND.

## Laboratory Indicators

Regarding laboratory tests, studies indicate the role of the de Ritis index (alanine aminotransaminase/aspartate aminotransaminase ratio). In studies on the impact of preoperative blood marker levels conducted on a group of 135 patients, it was noted that the elevated de Ritis index ≥ 1.3 is closely correlated with the presence of LNM (*p* = 0.0096) [93, 94]. Another tumor pathophysiology aspect of growing interest is systemic immune inflammation, which modulates metastasis and tumor invasion [95, 96]. Index of systemic inflammation (SII; neutrophil*thrombocyte/lymphocyte ratio) is an inexpensive tool validating tumor’s response to treatment and anticipating prognosis [97–100]. In UTUC, high SII values correlate with positive LVI, which affects OS, CSS, and PFS rates [100]. In a recent study, Kobayashi et al. developed models comprising SII > 520, ECOG-PS > 0, and ≥ cT3 as three preoperative risk scores, based on which patients can be classified as requiring LND or adjuvant ChT [101••].

## Genetic Markers

UTUC staging may hopefully include analyzing gene and transcript expression levels in the future. Among the genes studied by researchers is FBLN2, encoding fibulin 2 [90, 102–107]. Overexpression of FBLN2 is associated with poor DSS and MFS rates in both UTUC and UCB (*p* < 0.001 in both malignancies), and with higher stage tumors, LNM, and high mitotic activity [90]. Another investigated gene is PDK [108]. It has previously been associated with tumor aggressiveness, proliferation, and resistance to ChT in UCB [109, 110]. A study by Kuo et al. demonstrated that the expression of PDK3 influences the proliferation of UTUC through its involvement in DNA replication and repair processes. Researchers revealed that overexpression of PDK3 correlates with more advanced tumor stages, LNM, higher tumor grades, and increased mitotic index. Moreover, DSS and MFS outcomes were significantly worse in cases where PDK3 in-tumor expression levels were higher (*p* < 0.0001 for both) [111]. The cartilage oligomeric matrix protein (COMP) might serve as another negative prognostic factor for UTUC [112–115]. COMP overexpression is associated with advanced T stage, LNM, LVI, PNI, high histological grade, and high mitotic rate in UTUC [116]. The study by Li et al. also highlights the significance of the metallothionein 2A (MT2A) as a marker of tumor aggressiveness in both UTUC and UCB [117]. MT2A can serve as a prognostic factor for assessing the risk of tumor severity as it is significantly associated with high tumor stage, LNM, high tumor grade, LVI, and PNI [117].

Other potential genetic prognostic factors for nodal invasion involve the following: solute carrier family 14 member 1 (SLC14A1), ring finger protein 128 (RNF128), nuclear protein Ki67 (Ki67), insulin-like growth factor-binding protein-5 (IGFBP-5), forkhead box O3 (FOXO3A), human epidermal growth factor receptor 2 (ERBB2), chitinase 3-like-1 (CHI3L1), receptor tyrosine kinase–like orphan receptor 2 (ROR2), epidermal growth factor–containing fibulin-like extracellular matrix protein 1 (*EFEMP1*), stromal periostin (PN), trophoblast cell surface antigen 2 (Trop-2), B7 homolog 3 (B7-H3), B7 homolog 4 (B7-H4), nectin-4, polymerase I and transcript release factor (PTRF), GATA binding protein 3 (GATA3), tumor necrosis factor alpha-induced protein 6 (TNFAIP6), phosphatase and tensin homolog deleted on chromosome 10 (PTEN), human epidermal growth factor receptor 2 (HER2), E-cadherin, fibroblast growth factor 7 (FGF7), human antigen R (HuR), kisspeptins (KiSS-1), Rac1 small GTPase (Rac1) [118–141]. Table 4 presents an overview of genetic factors associated with nodal involvement in UTUC.

**Table 4:** Overview of genetic factors correlated with nodal metastasis in UTUC

Expression	Genetic factors
Overexpression	CHI3LI1, COMP, FBLN2, IGFBP-5, Ki-67, MT2A, PDK3, ERBB2, ROR2, EFEMP1, PN, B7-H4, nectin-4, PTRF, TNFAIP6, HER2, FGF7, HuR, Rac1
Underexpression	FOXO3A, RNF128, Trop-2, GATA3, PTEN, E-cadherin, KiSS-1

*CHI3LI1* chitinase 3-like-1, *COMP* cartilage oligomeric matrix protein, *FBLN2* fibulin 2, *IGFBP-5* insulin-like growth factor-binding protein-5, *Ki-67* nuclear protein Ki-67, *MT2A* metallothionein 2A, *PDK3* pyruvate dehydrogenase kinase-3, *ERBB2* human epidermal growth factor receptor 2, *ROR2* receptor tyrosine kinase–like orphan receptor 2, *EFEMP1* epidermal growth factor–containing fibulin-like extracellular matrix protein 1, *PN* stromal periostin, *B7-H4* B7 homolog 4, *PTRF* polymerase I and transcript release factor, *TNFAIP6* tumor necrosis factor alpha-induced protein 6, *HER2* human epidermal growth factor receptor 2, *FGF7* fibroblast growth factor 7, *HuR* human antigen R, *Rac1* Rac1 small GTPase, *FOXO3A* forkhead box O3, *RNF128* RING finger protein 128, *Trop-2* trophoblast cell surface antigen 2, *GATA3* GATA binding protein 3, *PTEN* phosphatase and tensin homolog deleted on chromosome 10, *KiSS-1* kisspeptins

## Imaging Tests

The currently available CT and positron emission tomography (PET) examinations are insufficient in accurately localizing LNM in UTUC [142, 143, 144•, 145]. This statement confirms the findings of a multicenter study conducted in 2023, which demonstrated through a retrospective analysis that conventional imaging exhibits limited sensitivity of 25% (95% CI 20; 31) in detecting LNM in UTUC [145]. Therefore, researchers are actively seeking markers and exploring methods to combine existing examinations to improve nodal staging. The literature suggests combining 18F-fluorodeoxyglucose PET with CT (18FDG-PET/CT) as one of the ways to detect LNM in UTUC and UCB in great detail [146•, 147–152]. While the study by Jensen et al. reported similar sensitivity and specificity between 18FDG-PET/CT and MRI in detecting LNM, subsequent research has demonstrated the superiority of the combined method over separate studies using MRI, CT, or PET [150, 152, 153]. The enhanced effectiveness of 18FDG-PET/CT stems from its ability to detect highly metabolic micrometastases, which are too small to be identified by CT alone (< 2.0 mm in largest dimension), thus improving the sensitivity of LN staging [149, 154]. However, 18FDG-PET/CT has low specificity, making it challenging to differentiate between inflammatory and metastatic LNs [149]. Several retrospective studies have investigated the use of this method for preoperative detection of LNM in UTUC and UCB [146•, 147]. In a 2020 study specifically evaluating preoperative detection of LNM, the 18FDG-PET/CT method exhibited a sensitivity of 82% and specificity of 84% [146•].

A 2020 systematic review, which included three retrospective studies on LNM detection in UTUC, reported sensitivities ranging from 82 to 95% and specificities ranging from 84 to 91%. These high percentages indicate the substantial prognostic value of 18FDG-PET/CT [149]. Furthermore, a study comparing combined method (18FDG-PET/CT) with CT in UCB for nodal staging revealed sensitivities of 78% and 44%, respectively [155]. The issue with the 18FDG-PET/CT technique is that 18FDG is excreted in the urine, which interferes with the interpretation of images of the bladder and nodal lesions near ureters [156].

The utilization of PET/CT with (11)C-choline, also known as choline PET/CT, is under consideration for diagnosing LNM in UTUC. A study conducted in 2014 demonstrated that patients with UTUC exhibited high choline uptake in the affected LNs [157].

In the study conducted by Polom et al., researchers aimed to detect sentinel lymph nodes (SLNs) by administering Technetium-99 m (99mTc) injection during ureterorenoscopy and evaluating the results through single-photon emission-computed tomography/computed tomography (SPECT/CT) lymphangiography. The findings of the study indicated that while it is theoretically possible to locate SLNs using this method, it proved to be highly challenging due to difficulties associated with injecting Technetium during the course of the study [158•].

There is ongoing research on labeled monoclonal antibodies and their use in the management of urothelial neoplasms [144•]. The use of girentuximab-labeled PET/CT (89Zr-TLX250) appears to be the most promising approach, given its established efficacy in guiding clinical evaluations of renal cell carcinoma and its possible utility in breast cancer staging. TLX250 is an antibody directed against carbonic anhydrase IX (CAIX), an enzyme showing high activity in urothelial cancer cells [159]. The ongoing phase I study is expected to answer the question of whether 89Zr-TLX250 allows efficient imaging of urothelial malignancies [155].

Imaging studies are currently being utilized as a contributing factor in the development of a preoperative evaluation protocol to determine the presence of LN metastases in UTUC. A study conducted last year demonstrated that by incorporating imaging and biopsy data such as stage, LVI, tumor size, and positive clinical LN status, it was possible to predict the probability of LN metastases in UTUC with an accuracy of 87.8% (AUC 0.878, corrected C-index 0.887) [160••].

## Various Surgical Approaches

Although RNU is the preferred surgical treatment for UTUC, it has drawbacks including the risk of decreased renal function and no guarantee of recurrence-free outcomes, leading to increasing interest in exploring less radical approaches [161–163]. These alternative options are minimally invasive or nephron-sparing methods. These approaches aim to minimize complications, preserve renal function, and effectively treat UTUC [162, 164].

## Nephron-Sparing Approach

Nephron-sparing approaches for UTUC include segmental ureterectomy (SU), ureterorenoscopy, or intraluminal therapy [162, 165, 166]. These approaches remove the tumor while preserving kidney function, resulting in a lower risk of kidney failure compared to RNU. Nephron-sparing methods are used for patients with small volume, noninvasive, and low-grade tumors [161, 162, 167]. Both the EAU and NCCN guidelines recommend nephron-sparing treatment as a viable alternative to RNU for low-risk UTUC patients. These approaches aim to achieve tumor control while minimizing complications associated with radical surgery [161, 162, 167].

These methods are particularly beneficial for patients with a solitary functioning kidney, bilateral disease, or chronic kidney disease. Clinicians with these techniques can customize treatment based on individual patient needs, ensuring optimal outcomes while reducing the risk of complications [168].

During ureterorenoscopic surgery or intraluminal therapy, LND is not feasible, ruling out their use in suspected LNMs [169]. An alternative for ureter-localized UTUC is SU with regional LND, even for high-risk cancer [170]. A 2022 meta-analysis indicates that the SU and RNU have similar RFS, PFS, CCS, and OS rates. However, accurate staging and precise diagnosis of UTUC are crucial for determining the suitability of SU. Meticulous patient selection is essential for maximizing the benefits associated with this method [162].

## Minimally Invasive Surgery

Minimally invasive surgery, including laparoscopic nephroureterectomy (LNU) and robotic-assisted nephroureterectomy (RRNU), offers advantages over traditional RNU such as shorter postoperative recovery, minimal blood loss, improved LND rates, and reduced short-term morbidity [164, 171–174]. Treatment outcomes are similar to classic RNU, with the benefit of lower perioperative mortality using robotic surgery (OR 0.7, 95% CI 0.53–0.91, *p* = 0.008). However, further studies are needed to confirm these findings as the meta-analysis that reported this conclusion had some heterogeneity among the studies (*I*^2^ = 50%) [171, 175].

Minimally invasive surgery, unlike the nephron-sparing approach, provides the option of LND for patients who require this procedure [171, 176]. Widely used robotic systems are the da Vinci Si® and Xi® [177••]. Studies show that the use of the Xi® is safe and can enable extensive LND without open surgery [171]. Preserving the integrity of the LNs during extraction, particularly in patients with pT3-T4 disease, is essential to minimize the potential dissemination of cancer cells [176]. Additional precautions during laparoscopic surgeries include avoiding entry into the urinary tract, preventing direct instrument-tumor contact, and using an endobag for tumor extraction [10].

A 2020 study analyzing data from three specialized centers using robotic techniques for treatment found no increased risk of tumor spread with these surgical methods. The extent of LND, whether a template LND or resection of only enlarged LNs, was determined by the surgeon. The study reported minimal perioperative mortality and no conversions to open surgery, suggesting that RRNU holds promise as a future treatment modality, even in advanced stages requiring LND [177••].

## Conclusions

Several prognostic factors for LN involvement in UTUC have been identified, but LND remains the only effective nodal staging tool. However, the therapeutic benefit of lymphadenectomy is still inconclusive. Mapping studies have contributed our understanding of LN drainage sites, redeveloping the anatomical scope of LND, and potentially enhancing patient survival. Nevertheless, further prospective multi-center studies are required to comprehensively assess the advantages and limitations of LND in UTUC.

## Data Availability

The authors confirm that the data supporting the findings of this study are available within the article and its supplementary materials.
